# Early Disseminated Lyme Carditis Inducing High-Degree Atrioventricular Block

**DOI:** 10.1155/2020/5309285

**Published:** 2020-06-03

**Authors:** Connor C. Kerndt, John A. Bills, Zaid J. Shareef, Alexander M. Balinski, Daniel F. Summers, Jose M. Tan

**Affiliations:** ^1^Department of Internal Medicine, Spectrum Health Butterworth Hospital, Grand Rapids, Michigan, USA; ^2^Michigan State University College of Osteopathic Medicine, East Lansing, Michigan, USA; ^3^Oakland University William Beaumont School of Medicine, Rochester, Michigan, USA; ^4^Department of Cardiovascular Disease, Spectrum Health Butterworth Hospital, Grand Rapids, Michigan, USA

## Abstract

Lyme disease is the most common tick-borne illness in the United States due to *Borrelia burgdorferi* infection. This case demonstrates a 20-year-old male patient presenting with complaints of annular skin rash, malaise, fever, and lightheadedness after significant outdoor exposure. Physical exam revealed multiple large targetoid lesions on the back and extremities. The rash had raised borders and centralized clearing consistent with erythema migrans chronicum. Electrocardiogram (ECG) revealed a high-degree atrioventricular (AV) block. The patient was started on intravenous ceftriaxone due to clinical suspicion for Lyme carditis. ELISA and Western blot tests were reactive for Lyme IgM and IgG, confirming the diagnosis. The AV block resolved by hospital day four and the patient was discharged with outpatient follow-up. Early identification of disease allowed for effective treatment with no adverse outcomes or sequelae.

## 1. Introduction

Lyme disease is the most common tick-borne illness in the United States [1]. Infection is caused by the spirochete *Borrelia burgdorferi* which is transmitted to humans via an Ixodes tick bite [2]. Lyme disease most commonly manifests during the late spring and summer months in the Northeastern United States and Wisconsin with exposure to wooded outdoor areas [3, 4]. Early intervention is critical to avoid the devastating sequelae of disseminated Lyme disease such as neurological impairment, chronic arthritis, and infection-induced heart block [2, 5, 6].

## 2. Case Presentation

A 20-year-old male patient with no prior medical history presented to the hospital with complaints of skin rash, malaise, and fever. The patient worked as a summer camp counselor and had recently been camping in Wisconsin. Several weeks after returning from a camping excursion, he noticed a nontender, nonpruritic annular rash on his arm with centralized clearing, absent of pain or pruritus. Three days later, he experienced excessive fatigue and accompanying fever (T-max 101°F). The patient presented to the camp clinic with a characteristic rash raising suspicion for Lyme disease. Given his age, symptomatology, recent outdoor exposure, and distinctive rash, a Suspicious Index in Lyme Carditis (SILC) score of 9 gave high suspicion for early Lyme carditis. The patient was sent to a tertiary care center for further evaluation and management.

**Figure 1:**
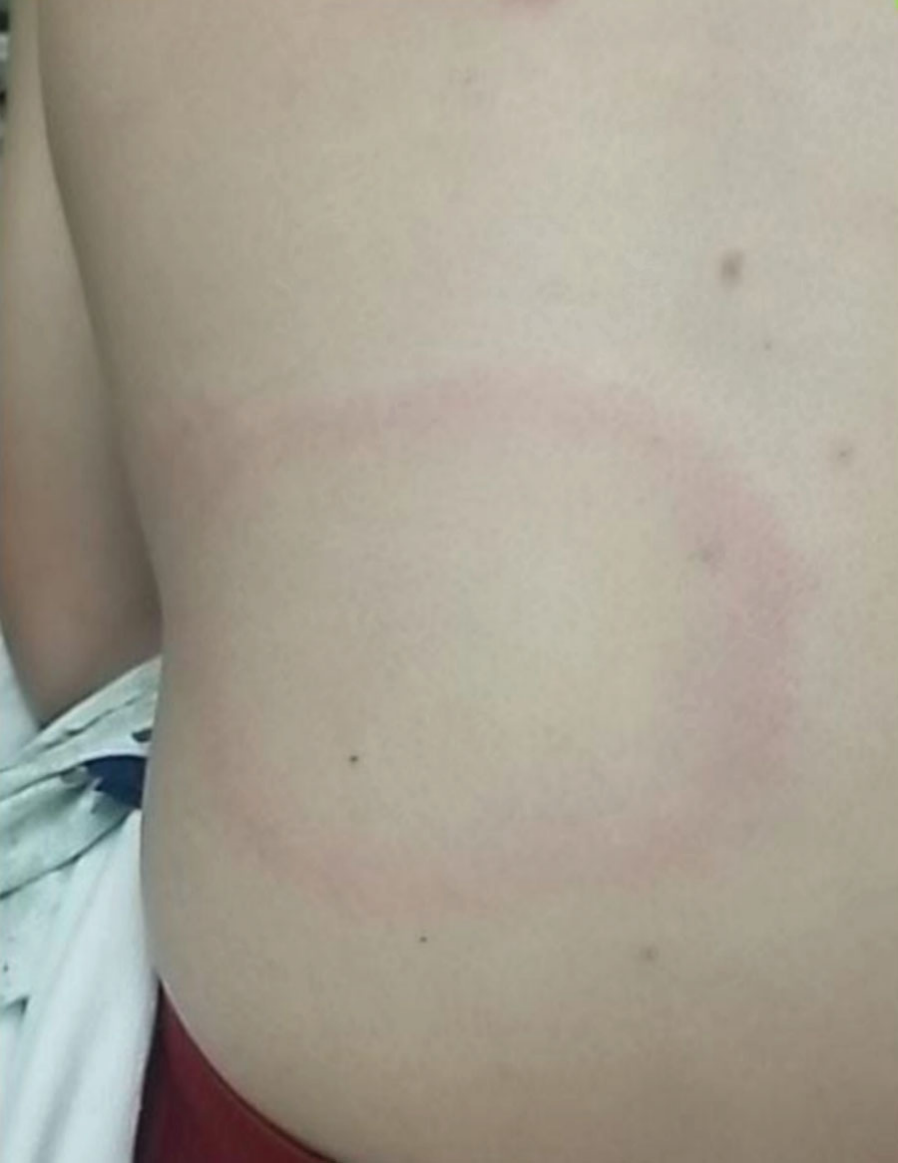
Dorsal targetoid lesion with centralized clearing (erythema migrans chronicum).

Upon admission, the patient developed intermittent symptomatic bradycardia with an average heart rate of 40 bpm one episode of severe bradycardia with a nadir of 15 bpm over a period of five seconds. The patient complained of accompanying generalized fatigue and intermittent lightheadedness. He denied complaints of arthralgia, myalgia, motor/sensory deficit, headache, altered mental status, or neck pain. Physical exam revealed multiple large targetoid lesions on the lower extremities, upper extremities, and back (Figure 1). The lesions had raised borders with centralized clearing consistent with erythema migrans chronicum.

**Figure 2:**
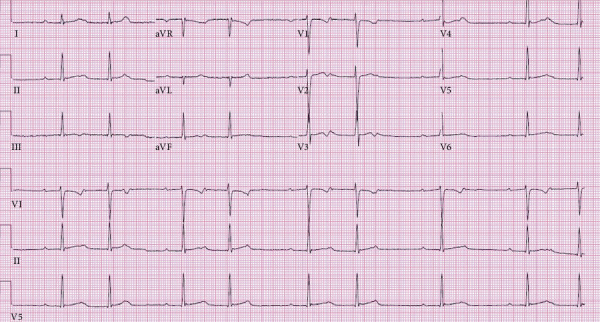
ECG demonstrating sinus rhythm with second-degree AV block (Mobitz Type I).

**Figure 3:**
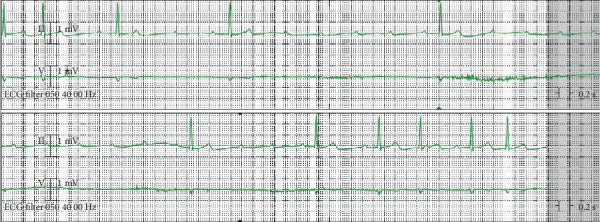
Telemetry demonstrating high-grade AV block.

Presenting electrocardiogram (ECG) revealed a second-degree atrioventricular (AV) block, Mobitz Type I (Figure 2). Inpatient telemetry demonstrated episodes of high-grade AV block (Figure 3). Transthoracic echocardiogram demonstrated a normal ejection fraction of 73% with no regional wall motion abnormalities. The valve anatomy and function were also normal. Antibiotic therapy was initiated with 2 g of intravenous (IV) ceftriaxone due to the characteristic presentation of Lyme carditis. Atropine and transcutaneous pacing were deferred due to relative clinical stability. Follow-up Lyme ELISA was reactive for Lyme IgM and IgG, confirmed by Western blot that showed reactivity of IgM to p23, p39, and p41. IgG was reactive to p18, p23, p39, p41, and p93 (Table 1). Given the ECG findings and serology, the patient was diagnosed with early disseminated Lyme carditis.

**Figure 4:**
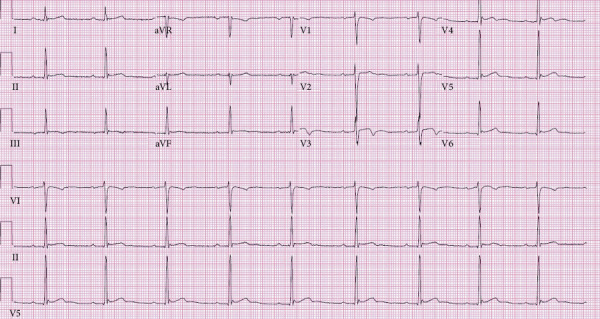
ECG demonstrating sinus bradycardia with first-degree AV block.

Intravenous ceftriaxone was initiated for 28 days, four days inpatient and 24 days outpatient via a peripherally inserted central catheter. After two days of therapy, the annular skin lesions resolved with coinciding resolution of fever and malaise. Heart block also progressively improved from high-grade AV block to second-degree AV block (Mobitz Type I). On the fourth day of treatment, the predominant rhythm was a first-degree AV block maintaining adequate PR interval control measured at 216 ms upon discharge (Figure 4). The patient was discharged on hospital day four. Follow-up appointments with electrophysiology and infectious disease services were arranged for complete resolution of his PR interval and further testing.

**Figure 5:**
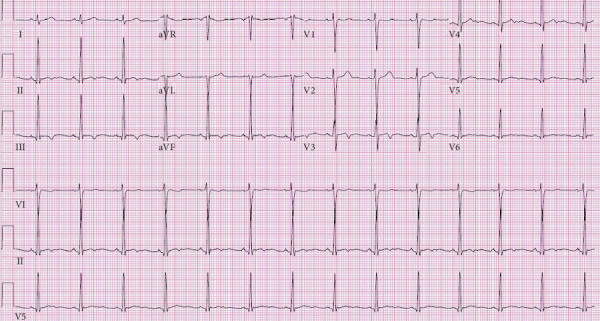
Follow-up ECG demonstrating normal sinus rhythm and resolution of AV block.

On follow-up, the patient reported complete resolution of symptoms and a return to baseline. He did not have any headache, joint pain, fatigue, presyncope, or syncope. Follow-up laboratory panel was also unremarkable with no significant derangement. Patient follow-up ECG showed a normal rate and sinus rhythm with complete resolution of the previous AV block (Figure 5).

## 3. Discussion

Lyme carditis is the most common vector-borne infection in Northeastern America, yet cardiac manifestations are uncommon [2, 7]. Early localized Lyme disease is caused by systemic infection of *Borrelia burgdorferi*, classically presenting as erythema migrans with or without constitutional symptoms [4]. If left untreated, late systemic disease can create cardiac complications such as reversible AV block, cardiomyopathy, and myocarditis due to spirochete infiltration of cardiac and pericardial structures [8]. Given the cardiac tissue irritation in Lyme carditis, a patient's ECG can exhibit marked derangement including ST-segment changes, t-wave inversion, repolarization abnormalities, and atrioventricular block [3, 9]. First-degree AV block is the most common cardiac arrhythmia of Lyme carditis, but some cases have demonstrated second-degree AV block, third-degree AV block, atrial fibrillation, tachyarrhythmia, and QT prolongation [3, 8–12]. Fluctuations between first-degree and second-degree conduction block have also been reported [2, 9], but first-degree block seldomly fluctuates to the level of complete heart block [13].

Clinical suspicion of early disseminated Lyme carditis is essential in patients presenting with new-onset high-degree AV block. The absence of appropriate treatment can result in progression of disease and worsening symptomatology [14]. Failure to recognize early Lyme carditis is associated with an increased need for permanent pacemaker implantation [14]. To help distinguish etiology of high-degree AV block, an evidence-based scoring system termed the “Suspicious Index in Lyme Carditis” was developed in 2018 [15]. The parameters contributing to this score incorporate common presentations of Lyme disease, including male sex, age less than 50 years, recent outdoor activity or endemic exposure, constitutional symptomology, known tick bite, and classical erythema migrans rash [15]. The cumulative score categorizes patients based on level of suspicion for Lyme disease as the etiologic cause of AV block [15].

Current guidelines for diagnosis and management of Lyme carditis suggest stratifying suspicion of Lyme-induced AV block into low, intermediate, and high-risk categories using an SILC score [10]. SILC scoring reports a sensitivity of nearly 92.9% for intermediate and high suspicion categories, making it a valuable screening tool for decreasing unnecessary laboratory testing and antibiotic use [15]. However, the failure of SILC to confer suspicion based on laboratory and histologic findings may warrant additional diagnostic workup outside of its recommendation [16]. After SILC scoring, individuals with low suspicion receive standard treatment for AV block and those with intermediate or high suspicion begin empiric IV antibiotic therapy and serologic testing [15]. Patients exhibiting asymptomatic bradycardia are managed with strict cardiac monitoring [10, 17], while symptomatic patients require continuous cardiac telemetry to evaluate the need for transcutaneous pacing or inotropic support due to the risk of ischemia or death with persistent high-grade AV block [13, 17–19].

In this case, the patient obtained a SILC score of 9 due to his status as a young male with classic symptomatology, outdoor activity, and pathognomonic erythema migrans rash. This score placed him within the category of high suspicion of early disseminated Lyme carditis. New-onset heart block in a young patient is exceptionally rare (1/15,000 cases) with widely variable etiologies [20–24]. Electrolyte abnormalities such as hyperkalemia, as well as thyroid dysfunction, may induce heart block and should be considered in these cases [4, 25, 26]. Without the pathognomonic rash, the differential diagnosis may have been significantly confounded in this patient. This emphasizes the importance of a thorough integumentary examination and exposure history in patients with heart block of unidentified etiology [20–24].

In the workup of this case, pending Lyme serology resulted in a comprehensive patient evaluation to assess other possible AV block etiologies. Transthoracic echocardiogram, complete blood count (CBC), complete metabolic panel (CMP), thyroid panel, and autoantibody testing were all conducted to rule out the etiology of noninfectious heart block in this patient. Systemic lupus erythematosus (SLE) was also considered [4], but the presence of erythema migrans and absence of renal and hematologic dysfunction were more consistent with Lyme carditis. Inpatient genetic testing was deemed unnecessary for this patient as genetic causes of AV block were ruled out due to the absence of previous personal and/or family cardiac history. Cardiac biopsy for evaluation of infiltrative disease was also deferred given the procedural risk and relative probability of Lyme etiology [4, 27, 28].

With greater than 95 percent specificity, ELISA antibody and Western blot serologic studies were utilized to establish a diagnosis of early disseminated Lyme carditis in this case. Based on the current guidelines, the patient was tested for Lyme serology and started on intravenous ceftriaxone [15, 29]. It is recommended that patients with negative serology proceed to standard high-degree AV block management, while patients with positive serology receive IV ceftriaxone for 10-14 days followed by oral antibiotics (preferably doxycycline) to complete a total course of 14-21 days of antibiotic therapy [10, 30]. For this patient, positive IgM and IgG serologic markers solidified a primary diagnosis of early disseminated Lyme carditis (Table 1) [31] and IV ceftriaxone was continued.

While the CDC recommends an antibiotic course of 14-21 days for Lyme carditis, other manifestations of Lyme disease follow protocols lasting up to 28 days and no clinical trials have been conducted to evaluate appropriate durations of antibiotic treatment [10, 18, 30, 32]. In this case, a 28-day course of ceftriaxone was considered appropriate to ensure clearance of Lyme infection. Furthermore, the patient was not transitioned to oral doxycycline due to a past history of adverse drug reaction [18]. Per current guidelines, the patient obtained a follow-up ECG demonstrating 1 : 1 conduction, but did not receive a stress ECG. This demonstrates a limitation in patient management, as follow-up stress testing is recommended to evaluate AV conduction in a noninvasive manner 4-6 weeks after initial resolution of AV block [10]. Stress ECG testing is critical for appropriate analysis of the point of Wenckebach which can demonstrate cardiac recovery and determine if permanent cardiac pacing, repeat stress testing, or routine ECG follow-up is indicated [17].

## 4. Conclusion

Lyme carditis is a known etiologic cause of cardiac conduction abnormalities, particularly AV block. SILC scoring can be a useful tool in the evaluation and treatment of suspected Lyme-induced heart block. Stratifying suspicion allows for indicated serologic testing and time-sensitive antibiotic treatment. While patients with early disseminated Lyme carditis carry a good prognosis, delayed management can result in long-term complications and poor cardiac outcomes.

## Figures and Tables

**Table 1:** Lyme serology results.

	Lyme IgG							Lyme IgM			
	WB	p18	p23	p39	p41	p45	p93	WB	p23	p39	p41
Serum	+	+	+	+	+	+	+	+	+	+	+
